# From BMI to TMI: revisiting adiposity and fitness assessment in young active adults through a historical and contemporary lens

**DOI:** 10.3389/fpubh.2025.1700684

**Published:** 2025-11-14

**Authors:** Onur Mutlu Yaşar, Veli Volkan Gürses, Ali Erdem Ciğerci, Erdal Bal, Yeliz Pehlivan, Mustafa Baş, Nedim Malkoç, Merve Bektaş, Gizem Başkaya, Sare Dündar, Ömür Fatih Karakullukçu, Hamza Küçük

**Affiliations:** 1 Faculty of Health Sciences, Izmir Demokrasi University, Izmir, Türkiye; 2 Faculty of Sport Sciences, Bandırma University, Balıkesir, Türkiye; 3 Faculty of Sports Sciences, Kastamonu University, Kastamonu, Türkiye; 4 Faculty of Life Sciences, University of Health Sciences, Istanbul, Türkiye; 5 Ministry of National Education, Ankara, Türkiye; 6 Yasar Dogu Faculty of Sport Sciences, Ondokuz Mayis University, Samsun, Türkiye

**Keywords:** physically active young adults, body fat, body mass index, hematology, triponderal mass index

## Abstract

**Background:**

Traditional reliance on body mass index (BMI) to assess adiposity may misclassify physically active individuals with high lean mass. The triponderal mass index (TMI) has emerged as a potentially more accurate alternative, but evidence in physically active populations is limited.

**Purpose:**

To examine the associations of TMI and BMI with bioimpedance-derived adiposity and selected hematological markers in physically active young adults.

**Methods:**

In this cross-sectional study, (*n* = 59) participants (male = 37, female = 22; age 22.63 ± 2.29 years) underwent anthropometry, whole-body bioimpedance and venous blood sampling. TMI and BMI were calculated, and associations with body fat percentage, hemoglobin, hematocrit, neutrophil-to-lymphocyte ratio (NLR), and platelet-to-lymphocyte ratio (PLR) were examined. Normality assumptions were checked; correlations and multiple linear regressions were computed (*α* = 0.05). Physical activity status followed WHO/ACSM guidelines (≥150 min·week^−1^ of moderate-to-vigorous activity).

**Results:**

TMI showed a stronger positive correlation with body fat percentage than BMI (*r* = 0.50, *p* < 0.001 vs. *r* = 0.38, *p* = 0.003). BMI was positively correlated with HGB (*r* = 0.32, *p* = 0.013) and HCT (*r* = 0.26, *p* = 0.046) and negatively correlated with NLR (*ρ* = −0.27, *p* = 0.041) and PLR (*r* = −0.30, *p* = 0.022). TMI was negatively correlated with NLR (*p* = −0.27, *p* = 0.039). In the multivariable model (predictors: BMI, TMI, HGB, HCT, NLR, PLR), HCT was a significant negative predictor of adiposity (*β* = −0.34, *p* = 0.021), whereas TMI showed a positive but marginally non-significant association (*β* = 0.25, *p* = 0.073). Assumptions and multicollinearity were acceptable.

**Conclusion:**

In physically active young adults, TMI relates more strongly than BMI to bioimpedance-derived adiposity and may aid field-based screening. However, athlete-level decisions should await confirmation in sport-specific, longitudinal studies using criterion methods.

## 1 Introduction

Accurate assessment of body composition is critical in sports science, as it influences athletic performance, training adaptation, and long-term health outcomes (1–11). Body mass index (BMI), calculated as body mass in kilograms divided by height in meters squared (kg/m^2^), is one of the most widely used indices for estimating adiposity in both clinical and young active adults settings (12–16). However, BMI does not distinguish between fat mass and lean body mass, which can lead to misclassification, particularly in physically active populations with higher muscle mass (17, 18). This limitation has prompted the exploration of alternative anthropometric measures that can more accurately reflect body fatness and metabolic risk in active people.

The triponderal mass index (TMI), calculated as body mass in kilograms divided by height cubed (kg/m^3^), has recently emerged as a potentially superior measure for estimating body fatness, especially in adolescents and young adults (19–23). Unlike BMI, TMI appears less influenced by variations in height and pubertal maturation, offering greater stability across growth stages (24–26). Studies have reported that TMI demonstrates stronger correlations with direct measures of body fat, such as dual-energy X-ray absorptiometry (DXA), compared to BMI in youth populations (27–29). BMI is widely used for population-level screening but may insufficiently capture adiposity at the individual level. TMI has shown superior validity in pediatric and adolescent cohorts and is theoretically less sensitive to height-related allometry. However, evidence in physically active young adults is limited. TMI may demonstrates stronger relationships than BMI with bioimpedance-derived adiposity and hematological markers in active university students.

In addition to anthropometric indices, hematological parameters—such as hemoglobin (HGB), hematocrit (HCT), leukocyte differentials, and platelet indices—are important indicators of oxygen transport, inflammation, immune status, and overall physiological readiness (30–35). These markers can provide valuable insight into the metabolic and performance-related status of physically active individuals (36–38). Despite their relevance, few studies have simultaneously examined TMI, BMI, and a comprehensive hematological profile in active populations, limiting our understanding of how these indices relate to systemic health markers beyond body fat estimation alone (39–41).

Recent evidence suggests that TMI may provide equal or greater sensitivity than BMI in detecting adverse health markers, yet the majority of studies have been conducted in sedentary or clinical populations (27, 42–44). The lack of research in young, physically active individuals represents a significant gap, especially considering that accurate non-invasive screening tools are crucial for physical monitoring, training optimization, and injury prevention (45, 46). Therefore, aiming to investigate the relationships between TMI, BMI, body fat percentage, and hematological parameters among physically active young adults, to determine which index more accurately reflects systemic risk factors and adiposity could be valuable. By directly comparing these two indices alongside a detailed blood profile, this research seeks to refine anthropometric screening methods for application in sports science and exercise medicine. BMI is widely applied for population-level screening but may insufficiently capture adiposity at the individual level. TMI (mass/height^3^) has shown superior validity in pediatric and adolescent cohorts and is theoretically less sensitive to height-related allometry. Yet, evidence in physically active young adults remains limited. Addressing this gap, we examined whether TMI demonstrates stronger associations than BMI with bioimpedance-derived adiposity and hematological markers in active university students, while avoiding direct generalization to competitive athletes. We aimed to (i) compare the magnitude of associations between TMI/BMI and body fat percentage, and (ii) explore relationships with HGB, HCT, NLR, PLR. We hypothesized that TMI would correlate more strongly with adiposity than BMI in physically active young adults.

## 2 Materials and methods

### 2.1 Study design and setting

This cross-sectional, observational study was conducted at the Department of Exercise and Sport Sciences, Faculty of Life Sciences, Health Sciences University, Turkey. A stratified, multi-stage probability sampling method was utilized to ensure a representative distribution of participants based on sex and age among physically active individuals. The study protocol was approved by the Non-Interventional Clinical Research Ethics Committee of Health Sciences University (Approval No: 2025–2/2–27). All participants were informed about the aims and procedures of the study and provided written informed consent prior to participation. The study adhered to the ethical principles outlined in the Declaration of Helsinki.

### 2.2 Participants

We analyzed 59 physically active students (37 men, 22 women; age 22.63 ± 2.29 years). Mean height was 171.78 ± 8.16 cm, BMI 22.78 ± 2.91 kg·m^−2^, and TMI 13.28 ± 1.76 kg·m^−3^. Mean body fat percentage was 18.45 ± 3.81%. Hemoglobin (HGB) and hematocrit (HCT) were 14.95 ± 2.03 g·dL^−1^ and 44.85 ± 5.04%, respectively. Inclusion criteria included engaging in physical activity on ≥3 days/week, meeting WHO/ACSM guidelines (150–300 min/week of moderate-intensity, or 75–150 min/week of vigorous-intensity aerobic activity, or an equivalent combination), non-smoking status, no chronic diseases or regular medication use, and absence of endocrine or metabolic disorders. Physical activity status was self-reported and verified by a brief activity checklist. Exclusion criteria involved menstruation, acute infection, or inflammatory disease within 10 days prior to testing; leukocyte count >15,000/mm^3^; or eosinophil percentage >10% following blood analysis. Participants were instructed to abstain from food and caffeine for 10 h prior to measurements.

**Table:** 

Inclusion criteria	Exclusion criteria
Aged between 18–25 years	Acute infection or inflammatory condition within the past 10 days
Engaging in ≥3 days/week of physical activity (meeting the WHO/ACSM range)	Current medication use that may influence hematological status
Non-smoker	Diagnosed chronic disease (e.g., diabetes, anemia, thyroid dysfunction)
No history of metabolic or endocrine disorders	Menstruation during testing (for female participants)
Provided informed consent	WBC > 15,000/mm^3^ or eosinophil percentage > 10%
Enrolled as a university student	Incomplete anthropometric or blood measurements

### 2.3 Anthropometric assessments

Body height was measured to the nearest 0.1 cm using a stadiometer (Seca 213, Seca GmbH & Co. KG, Hamburg, Germany), with participants standing barefoot in anatomical position. Body mass and body fat percentage were assessed using a validated bioelectrical impedance analyzer (Tanita BC-545, Tanita Corp., Tokyo, Japan), with participants wearing light clothing and having emptied their bladder. Body Mass Index (BMI) was calculated using the standard formula (kg/m^2^), while Triponderal Mass Index (TMI) was derived as body mass (kg) divided by height cubed (m^3^). Waist circumference was measured at the midpoint between the lower costal margin and iliac crest using a non-elastic tape measure (Seca 203, Seca GmbH & Co. KG, Hamburg, Germany). Thigh circumference was measured at the mid-point between the trochanterion and tibiale laterale on the dominant leg.

### 2.4 Hematological assessments

Fasting venous blood samples were collected by a certified nurse from the antecubital vein using standard sterile techniques into EDTA-coated (purple-top) and serum-separation (yellow-top) vacutainer tubes (BD Vacutainer). Tourniquet application was limited to <60 s to avoid hemoconcentration artifacts. Each tube was gently inverted 6–8 times post-collection to ensure homogenous mixing with anticoagulants or clot activators. Samples were transported to the laboratory within 1 h and processed the same day. Hematological parameters, including red blood cell count (RBC), white blood cell count (WBC), hemoglobin (HGB), hematocrit (HCT), and platelet count (PLT), were analyzed using an automated hematology analyzer (Sysmex XN-Series, Sysmex Corp., Kobe, Japan). All procedures and analyses were supervised by a certified clinical biochemist.

### 2.5 Dependent and independent variables

The independent variables included BMI and TMI. The dependent variables consisted of hematological markers (RBC, WBC, HGB, HCT, PLT) and body fat percentage as assessed via bioelectrical impedance analysis. Demographic information age, lifestyle characteristics (exercise frequency, dietary habits), and anthropometric data were collected through structured questionnaires and direct measurements.

### 2.6 Standardization and quality control

All anthropometric and blood sampling procedures were conducted in the morning (between 08:00–10:00) to minimize diurnal variation. Instruments were calibrated before each testing session, and all measurements were performed by experienced personnel to ensure inter-rater reliability. A subset of measurements was repeated to assess test–retest consistency, with intra-class correlation coefficients (ICC) calculated to confirm reliability (ICC > 0.85 for all measures).

### 2.7 Statistical analysis

Statistical analyses were performed using IBM SPSS Statistics version 26.0 (IBM Corp., Armonk, NY, USA). Descriptive statistics were reported as mean ± standard deviation (SD) for continuous variables and frequencies (n, %) for categorical data. Normality of distribution was assessed using the Kolmogorov–Smirnov test. Between-group comparisons were conducted using independent t-tests for normally distributed variables and Mann–Whitney U tests for non-normal data. Bivariate correlations between BMI, TMI, body fat percentage, and hematological markers were analyzed using Pearson’s or Spearman’s correlation coefficients, depending on distributional assumptions. Multiple linear regression models were built to predict body fat percentage from BMI, TMI, and hematological markers (HGB, HCT, NLR, PLR). Sex was not included as a covariate to preserve model parsimony and because preliminary checks indicated no material improvement in model fit (Δ*R*^2^ < 0.01) and potential collinearity concerns. Multicollinearity was assessed via variance inflation factor (VIF) (threshold < 10); residual diagnostics confirmed linearity, homoscedasticity, and normality. The coefficient of determination (*R*^2^), standardized beta coefficients, *p*-values, and 95% confidence intervals were reported for each predictor. Significance was set at *p* < 0.05 for all analyses. *A priori* power analysis using G*Power 3.1 determined a minimum sample size of 59 participants to detect a moderate correlation (*r* = 0.35) with 80% power at *α* = 0.05. Effect sizes were interpreted as small (*d* = 0.2), medium (*d* = 0.5), or large (*d* = 0.8), and statistical significance was set at *p* < 0.05. All tests were two-tailed.

## 3 Results

### 3.1 Participant characteristics

Descriptive characteristics of the study sample are presented in Table 1. We analyzed 59 physically active students (34 men, 25 women; age 22.63 ± 2.29 years). Mean height was 171.78 ± 8.16 cm, BMI 22.78 ± 2.91 kg·m^−2^, and TMI 13.28 ± 1.76 kg·m^−3^. Mean body fat percentage was 18.45 ± 3.81%. WBC averaged 7.49 ± 1.87 × 10^3^·μL^−1^, PLT averaged 269.22 ± 52.88 × 10^3^·μL^−1^, and NEUT averaged 4.39 ± 1.42 × 10^3^·μL^−1^. The mean neutrophil-to-lymphocyte ratio (NLR) was 2.10 ± 1.05, and the mean platelet-to-lymphocyte ratio (PLR) was 127.33 ± 41.38. The distributions of WBC, PLT, and NEUT did not deviate from normality, whereas NLR deviated; accordingly, Spearman’s *ρ* was used for NLR in correlation analyses. BMI, TMI, body fat percentage, HGB, and HCT did not deviate significantly from normality (*p* > 0.05).

**Table 1:** Descriptive statistics of study variables (*n* = 59).

Variable	Mean	Sd.	95% Cl Lower	95% Cl Upper	Min.	Max.
Age (years)	22,63	2,28	22,05	23,21	19	30
Height	171.78	8.16	169.65	173.91	173.91	152.0
Weight	67.47	11.31	64.53	70.42	47.2	90.00
BMI (kg/m^2^)	22.78	2.91	22.02	23.54	16.99	28.67
TMI (kg/m^3^)	13.28	1.76	12,83	13,74	9.79	17.92
Body Fat (%)	18.45	3.81	17,46	19,44	10.80	25.40
HGB (g/dL)	14.95	2.03	14,42	15,48	8.20	19.70
HCT (%)	44.85	5.04	43,54	46,17	29.00	56.70
WBC (10^3^/μL)	7.49	1.87	7,0	7,97	3.74	14.10
PLT (10^3^/μL)	269.22	52.88	255,44	283,00	167.00	410.00
NEUT (10^3^/μL)	4.39	1.42	4,02	4,76	1.40	9.10
NLR	2.10	1.05	1,82	2,37	0.76	6.78
PLR	127.33	41.38	116,54	138,11	58.55	243.88

M = Mean; SD = Standard deviation; 95% CI = 95% confidence interval for the mean; Min = Minimum; Max = Maximum. BMI = Body Mass Index; TMI = Triponderal Mass Index; HGB = Hemoglobin; HCT = Hematocrit; WBC = White Blood Cells; PLT = Platelets; NEUT = Neutrophils; NLR = Neutrophil-to-Lymphocyte Ratio; PLR = Platelet-to-Lymphocyte Ratio.

### 3.2 Sex-based comparisons

As expected, males exhibited higher oxygen-carrying indices, whereas females demonstrated greater adiposity. Hemoglobin (HGB) was significantly higher in males compared with females (16.09 vs. 14.49 g·dL^−1^; *p* = 0.001), as was hematocrit (HCT) (47.35 vs. 43.58%; *p* = 0.004). Percent body fat was markedly higher in females (19.85 vs. 13.56%; p = 0.001). Body mass index (BMI) was modestly higher in males (23.41 vs. 21.94 kg·m^−2^), showing a trend toward significance (*p* = 0.057). Platelet-to-lymphocyte ratio (PLR) tended to be higher in females (134.7 vs. 120.2; *p* = 0.062), whereas neutrophil-to-lymphocyte ratio (NLR) did not differ significantly between sexes (*p* = 0.570).

### 3.3 Correlation analysis

**Figure 1:**
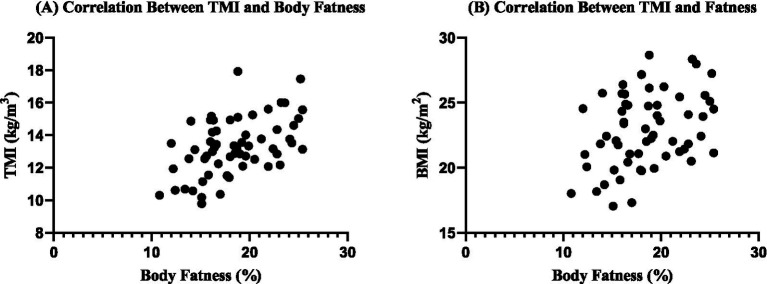
Bivariate correlation analysis of body fatness with BMI and TMI. Scatterplot matrix showing pairwise relationships between variables. Pearson’s correlation coefficients (*r*) with 95% confidence intervals. Scatterplots illustrating the correlations between body fat percentage and anthropometric indices. **(A)** Triponderal Mass Index (TMI, kg/m^3^) demonstrated a moderate positive association with body fat percentage, indicating higher TMI values correspond to greater adiposity. **(B)** Body Mass Index (BMI, kg/m^2^) also showed a positive relationship with body fat percentage, though the association appeared weaker compared to TMI. These findings support the potential utility of TMI as a more sensitive indicator of adiposity in physically active individuals.

Bivariate correlation analyses (Table 2) revealed that BMI was positively correlated with body fat percentage (*r* = 0.38, *p* = 0.003), HGB (*r* = 0.32, *p* = 0.013), and HCT (*r* = 0.26, *p* = 0.046). Conversely, BMI showed significant negative correlations with NLR (*r*ₛ = −0.27, *p* = 0.041) and PLR (*r* = −0.30, *p* = 0.022). TMI demonstrated a stronger positive correlation with body fat percentage (*r* = 0.50, *p* < 0.001) compared to BMI, while also exhibiting a negative correlation with NLR (*r*ₛ = −0.27, *p* = 0.039). No significant associations were observed between either BMI or TMI and WBC, PLT, or NEUT counts (Figure 1).

**Table 2:** Bivariate correlations between BMI, TMI, and hematological variables.

**Target**	**Variable**			
	BMI		TMI	
	***r***	***p***	***r***	***p***
Body fat %	0.38	0.003**	0.50	<0.001***
HGB	0.32	0.013*	0.15	0.244
HCT	0.26	0.046*	0.08	0.550
WBC	0.08	0.556	0.03	0.834
PLT	0.08	0.558	0.16	0.220
NEUT	−0.08	0.541	−0.12	0.384
NLR	−0.27	0.041*	−0.27	0.039*
PLR	−0.30	0.022*	−0.22	0.097

*r* = Pearson’s correlation coefficient; p = Significance level. *p* < 0.05 is considered statistically significant (*p* < 0.05 = *, *p* < 0.01 = **, *p* < 0.001 = ***). Positive *r* values indicate a direct relationship, whereas negative r values indicate an inverse relationship between variables.

### 3.4 Multiple regression analysis

A multiple linear regression model was conducted to examine the predictive capacity of BMI, TMI, HGB, HCT, NLR, and PLR for body fat percentage (Table 3). The overall model was statistically significant, *F*(6, 52) = 6.85, *p* < 0.001, explaining 44.1% of the variance in body fat percentage (Adj. *R*^2^ = 0.377). Among the predictors, HCT emerged as a significant negative predictor (*β* = −0.34, *p* = 0.021), indicating that lower hematocrit values were associated with higher body fat percentages. TMI showed a positive but marginally non-significant association (*β* = 0.25, *p* = 0.073). BMI, HGB, NLR, and PLR were not significant predictors when controlling for other variables. Multicollinearity diagnostics indicated acceptable variance inflation factor (VIF) values for all predictors, and residual plots confirmed that the assumptions of linearity, homoscedasticity, and normality were met.

**Table 3:** Multiple linear regression predicting body fat percentage.

Predictor	*B*	SE	*β*	*t*	*p*	95% CI LL	95% CI UL
Constant	22.95	6.42	—	3.57	0.001	10.06	35.84
BMI	−0.16	0.43	−0.06	−0.37	0.716	−1.03	1.25
TMI	1.28	0.70	0.25	1.83	0.073	−0.12	2.69
HGB	1.44	0.91	0.22	1.58	0.120	−0.39	3.28
HCT	−0.87	0.36	−0.34	−2.39	0.021	−1.60	−0.14
NLR	0.36	0.52	0.10	0.69	0.493	−0.69	1.41
PLR	−0.01	0.02	−0.09	−0.70	0.484	−0.04	0.02

Model fit: *R*^2^ = 0.441, Adj. *R*^2^ = 0.377, *F*(6, 52) = 6.85, *p* < 0.001. B = Unstandardized regression coefficient; SE = Standard error; β = Standardized beta coefficient; t = t-statistic; p = Significance level; 95% CI LL = Lower limit of 95% confidence interval; 95% CI UL = Upper limit of 95% confidence interval. *p* < 0.05 is considered statistically significant. All tests are two-tailed.

## 4 Discussion

This study examined how two low-cost anthropometric indices BMI and TMI relate to hematological markers of oxygen-carrying capacity and low-grade inflammation in physically active young adults. The findings suggest that TMI is a more precise anthropometric measure than BMI for estimating adiposity in active young populations and that integrating simple hematological markers particularly hematocrit (HCT) can provide additional physiological context in this group. Three findings stand out: (i) TMI correlated more strongly than BMI with bioimpedance-derived body fat percentage; (ii) BMI related positively to HGB/HCT, whereas both BMI and TMI related inversely, albeit modestly, to leukocyte-derived inflammatory ratios (NLR, PLR); and (iii) in multivariable modeling, lower HCT independently predicted higher adiposity after adjustment for anthropometry and inflammatory ratios. The HCT–adiposity association should be interpreted cautiously because it may partly reflect plasma-volume/hydration effects; mechanistic inference is further limited by the absence of ferritin and CRP. These results extend prior pediatric/adolescent evidence for TMI to an active young-adult cohort assessed under standardized conditions, but they should not be generalized to competitive athletes without sport-specific, longitudinal confirmation using criterion methods like DXA (47). In line with previous work showing that TMI can outperform BMI for estimating adiposity in youth and young adults, our data support its field-based screening utility while highlighting the need for sport-specific validation (3, 8, 31). These observations accord with recent reports that TMI better aligns with DXA-derived adiposity than BMI in non-clinical cohorts (28, 28, 48).

Below, we briefly position these findings within current evidence and outline practical implications for field based screening in physically active young adults. A central limitation of BMI in sports science is its inability to distinguish between lean and fat mass, often misclassifying muscular young active adults as overweight or obese despite low body fat levels (20). This misclassification risk is especially pronounced in adolescents and young adults engaged in resistance or mixed training, where increases in lean mass can inflate BMI without changes in fatness. By scaling mass to height cubed rather than squared, TMI more closely approximates body volume, thereby aligning more strongly with actual adiposity (22, 23). The closer coupling of TMI with adiposity aligns with a growing body of work reporting that height-cubed scaling better captures volumetric body size and fatness in populations where lean mass varies substantially (e.g., adolescents, young active adults, and active adults). In pediatric and youth cohorts, TMI has consistently outperformed BMI for classifying excess adiposity and tracking fat mass against criterion methods (22, 23). More recently, studies in sport and exercise settings have shown that TMI is less biased by muscularity and shows stronger correlations with DXA- or BIA-derived fat percentage than BMI, particularly when BMI lies in the “normal–overweight” band where misclassification risk is highest (20, 28, 47). The present results replicate earlier findings from youth and adolescent samples (27, 47) in an active young adult population, confirming that TMI correlates more robustly with bioimpedance-derived body fat percentage than BMI. For sports practitioners, this means that TMI can detect subtle changes in fatness even during periods of lean mass gain such as pre-season hypertrophy phases thereby improving the accuracy of nutritional and training adjustments. Our findings replicate this pattern in a physically active sample, supporting the practical recommendation to compute TMI alongside BMI during field screenings when DXA is unavailable.

Second, the hematology profile we observed differs from the adiposity-inflammation coupling often reported in sedentary or clinical cohorts. In those populations, higher adiposity is typically associated with elevated WBC counts and higher NLR/PLR, reflecting chronic low-grade inflammation (49, 50). In contrast, neither BMI nor TMI related to crude leukocyte or platelet counts here, and both indices showed small inverse relations with NLR/PLR. Two explanations are plausible. One is selection: these participants were physically active, a phenotype commonly characterized by lower basal inflammatory tone and favorable leukocyte trafficking at rest (51, 52). The second is range restriction: our cohort exhibited a relatively narrow, “healthy” BMI/TMI spectrum, which can attenuate or even invert associations that are monotonic in more heterogeneous samples. Together, these observations suggest that NLR/PLR may be less discriminative as risk markers in healthy active adults than in clinical screening, and that training load, fitness, and recovery status should be considered when interpreting immune-hematological ratios in fitness settings. In performance settings, precise body composition monitoring informs training load management. TMI is cost-effective and reduce the number of false positives for overweight classification in sports focused on strength and power. Furthermore, it has the capacity to discern bona fide alterations in adiposity that may not be fully apparent when using BMI, particularly with reference to seasonal fluctuations in muscle mass.

Third, the independent, negative association between HCT and adiposity in the multivariable model is physiologically coherent and has been reported in exercise studies when plasma volume status is considered. Greater adiposity especially in aerobically trained or habitually active individuals can co-occur with expanded plasma volume, producing lower HCT at a given red-cell mass (53, 54). Menstrual status and iron availability may also contribute to lower HCT among women with higher fat percentage, though we did not measure ferritin or hepcidin to test this pathway. Notably, HGB did not remain significant after adjustment, consistent with the notion that volume expansion (affecting HCT more directly) rather than reduced red-cell mass explains the signal. Practically, this underscores the value of pairing simple anthropometry with basic hematology (and, where feasible, ferritin/CRP) to contextualize field estimates of fatness in active populations.

Sex differences followed expected biological patterns higher HGB/HCT in men, higher fat percentage in women echoing recent sport-science reports and reinforcing the need for sex-aware reference frames when profiling young active adults and active adults (27, 28). Importantly, we did not find sex differences in NLR, again congruent with data suggesting training status narrows baseline inflammatory disparities (52).

BMI has long been criticized for its inability to differentiate between lean mass and fat mass, particularly in athletic populations where muscle mass is high. In such cases, young active adults with optimal or low body fat levels may still fall into the “overweight” BMI category [(20); Ode et al., 2007]. TMI, by scaling body mass to height cubed rather than squared, appears to reduce this bias and may provide a more volumetrically accurate estimate of body size (22, 23).

Our findings align with studies in adolescent and young active adults where TMI demonstrated stronger correlations with DXA-measured body fat percentage than BMI, particularly in sports emphasizing lean mass (e.g., sprinting, rowing, gymnastics) (27, 47). The advantage is especially relevant in youth sports development programs, where early detection of unhealthy fat gain or loss can inform training and nutrition adjustments without the misclassification risks inherent in BMI.

In high performance environments, anthropometric screening needs to be both accurate and feasible. While gold standard methods like DXA or hydrostatic weighing are ideal, they are not always accessible due to cost, portability, or scheduling constraints. TMI requires only body mass and height, making it: Field friendly: Easily measured on site at training facilities or competitions. Cost effective: No expensive devices are required, which is important for grassroots and youth programs. Sensitive in lean populations: More robust against the muscularity related inflation seen with BMI. Applicable in longitudinal tracking; Suitable for monitoring changes over a season or training cycle without specialized equipment.

For young active adults, TMI can help coaches and sports scientists: Differentiate training effects: Detect body composition changes that BMI might obscure, particularly when increases in lean mass are expected. Flag risk profiles: Identify early signs of excessive fat accumulation that could affect power-to-weight ratio, agility, or endurance. Support return to play protocols: Track body composition recovery after injury or illness, where muscle loss and fat gain may occur.

The inverse or null associations observed between adiposity indices and inflammatory markers (NLR, PLR) suggest that regular physical activity modulates baseline immune function. Active young adults generally exhibit lower resting inflammatory status and more favorable leukocyte distribution patterns than sedentary peers (51, 52). From a sports performance perspective, low systemic inflammation supports recovery, reduces injury risk, and may positively influence aerobic capacity. Interestingly, HCT emerged as an independent negative predictor of body fat percentage in our regression models. This may reflect plasma volume expansion, a common adaptation to endurance training rather than reduced red-cell mass (54). For coaches, monitoring HCT alongside anthropometric measures could provide additional context for athlete readiness and hydration status.

### 4.1 Practical applications in fitness monitoring

For field based screening in active student settings, TMI may provide a more informative anthropometric proxy of adiposity than BMI. In routine wellness checks, preseason evaluations, or campus health programs, incorporating TMI alongside BMI can help (i) flag individuals with potentially higher adiposity despite “normal” BMI, and (ii) monitor changes during lifestyle or training interventions. Until validated against criterion methods in sport-specific cohorts, athlete-level prescription or selection decisions should not rely on TMI alone; decisions should integrate performance tests, body composition by reference methods (e.g., DXA when feasible), and clinical judgment. This study adds to the growing evidence that TMI is not just a pediatric or epidemiological tool but also relevant in contexts particularly for young and active adults. While BMI remains widely used for its simplicity and historical inertia, integrating TMI into athlete health monitoring could enhance accuracy and prevent misinterpretation of muscular young active adults as “overweight.”

### 4.2 Limitations and future directions

This single-center study included a modest, homogeneous sample (*n* = 59) of physically active university students and employed a cross-sectional design. Adiposity was estimated via bioimpedance rather than a criterion method (e.g., DXA). Iron-status and inflammatory markers (e.g., ferritin, CRP) were not collected, which limits mechanistic interpretation of the observed HCT–adiposity association. Although physical activity status followed WHO/ACSM guidelines, self-report may introduce misclassification.

Validation of TMI against criterion methods is warranted in sport-specific cohorts, alongside longitudinal designs to test responsiveness to training-induced changes. Incorporating broader biochemical profiling (iron status, inflammation) and comparing sedentary, older, and youth groups would refine external validity and clarify physiological mechanisms.

## 5 Conclusion

In physically active young adults TMI shows stronger associations with bioimpedance-derived body fat than BMI and may aid field-based screening. Given the modest, homogeneous sample and the use of bioimpedance rather than a criterion method, confirmation in larger, sport-specific, and longitudinal studies—ideally including reference techniques (e.g., DXA) and broader hematological profiling—is recommended before informing athlete-level practice. Coupling TMI with simple hematological markers like HCT can enrich physical activity monitoring, inform individualized training, and reduce the risk of misclassification inherent in BMI. For health-related sport and exercise practitioners, this represents a low-cost, high-value upgrade to existing screening tools.

## Data Availability

The raw data supporting the conclusions of this article will be made available by the authors, without undue reservation.
